# Supplementary material for: Characterization of patients with advanced chronic pancreatitis using natural language processing of radiology reports
Source: PLoS One. 2020 Aug 19;15(8):e0236817. doi: 10.1371/journal.pone.0236817 (PMC7437899; doi:10.1371/journal.pone.0236817)
Supplement: S1 Appendix — (DOCX) [file pone.0236817.s001.docx]

**NLP Development**

The detailed steps are presented below.

1. Excluding sentences that belong to following scenarios:
   1. Sentences indicating diagnostic for organ sections other than pancreas, such as “liver:”, “biliary:”, “billary tree:”, “gallbladder:”, “kidney:”, “spleen:” or “bile duct:”.
   2. Sentences that are general information like “procedure:”, or “check for ”which described the standard procedure or the purpose for the imaging study, or like “history:”, “clinical information:” which are historical and clinical background for the imaging study.
   3. Sentences that are for patient instruction purpose like “discuss with the patient the fact that”.
   4. Sentences talking about hypothesis instead of observation/fact, such as starting with “if”, “whether”.
2. The pattern regular expressions were constructed to identify the compiled feature terms, modifier terms and the exclusion terms listed in Supplementary Table 1. Because our study’s interest was to identify pancreas-related features rather than those of other organs, the string “pancreas” was required to appear as part of the sentences. For the search of “ductal dilatation”, however, the phrase “pancreatic duct” was needed instead because this specific feature focuses on pancreatic (or main) duct.
3. Next, the relationship between a feature and a modifier was determined. If there was a modifier term associated with the feature term such as “dilation”, the number of words between these two terms (distance) was calculated. If the distance was larger than *10*, the NLP process stopped and moved to the next sentence.
4. The relationship between the detected feature and its related organ was then determined. If a pancreas-related term was identified within the sentence (either before or after the feature term) and organ term other than pancreas (i.e., “liver”, “renal”, “kidney”) was also detected between the feature term and the pancreas term, the NLP process stopped and moved to the next sentence with the assumption that the feature was associated with the other organ rather than with pancreas. For example, “pancreatic duct stents are in place and the intrahepatic bile ducts remain mildly dilated”. In this example, the feature of “dilatation” was in reference to the intrahepatic bile duct instead of the pancreatic duct.
5. Finally, if no pancreas-related term was found in a sentence, we extended the search to the sentences before and after the index sentence to look for the pancreas-related term. If the extended search did not find a pancreas-related term, the NLP stopped and moved to the next sentence.
6. For each feature, we labeled the status as “Definite”, “Probable” and “No” by implementing the negation algorithm in contextNLP/NegEx [1,2] . The lexical terms and negation rules in the contextNLP/NegEx were enhanced from the training process. For a given ascertained feature, the contextNLP searched all occurrences of the feature term within the sentence and ascertained the status of each occurrence with the outputs consisting of three categories: feature condition (positive, negative or unknown), certainty (yes or no) and temporality (new or old/history). For all the features we studied except for atrophy and calcification, the sentence level status was classified as “Definite” (feature condition=positive, certainty=yes, temporality=new), “Probable” (feature condition=positive, certainty=no, temporality=new), or “No” (the rest). Because atrophy and calcification usually persisted once they had presented, the classification was only based on feature condition and certainly, regardless of the value of temporality. Finally, the status was defaulted to “No” for these sentences in which the feature was not identified or the process was stopped in earlier steps.

The classified sentence-level results within same imaging report are combined into report-level and patient-level results for each imaging feature according to the following hierarchy.

Report-level:

1. “Definite”: if at least one sentence-level result within the report was “Definite”.
2. “Probable”: if at least one sentence-level result within the report was “Probable”.
3. “No”: If none of above sentence-level results within the report was detected.

Patient-level:

1. “Definite”: If the latest report-level result within the study period was “Definite”.
2. “Probable”: If at least one report-level results within the study period was “Probable”.
3. “No”: If none of above report-level results within the study period was detected.

**NLP Performance evaluation at the report level**

The results from manual chart review served as the reference standard to evaluate the performance of the computerized algorithm. The discrepancy between manual review and NLP algorithm results were fully adjudicated by the experienced gastroenterologist. For each imaging feature, the numbers of true positive (TP, both manual review and NLP classified as “Definite” or “Probable”) , false positive (FP, NLP classified as “Definite” and manual review classified as either “No” or “probable”, or NLP classified as “Probable” and chart review classified as “No”), true negative (TN, both manual review and NLP classified as “No”), and false negative (FN, manual review classified as “Definite” and NLP classified as either “No” or “Probable”, or manual review classified as “Probable” and NLP classified as “No”) cases were used to estimate the sensitivity, specificity, positive predictive value (PPV), negative predictive value (NPV), the overall accurate measure F-score. Sensitivity (recall) was defined as the number of TP divided by the total number of reports (TP+FN) ascertained by the manual review with corresponding imaging feature (“Definite” or “Probable”). Specificity was defined as the number of TN divided by the total of number of reports (FP+TN) ascertained by the manual review without corresponding imaging feature (‘No”) . PPV (precision) was defined as the number of TP divided by the total number of reports (TP+FP) identified by the computerized algorithm with imaging feature (“Definite” or “Probable”). NPV was defined as the number of TN divided by the total number of reports (TN+FN) identified by the computerized algorithm without imaging feature (“no”). The F-score was calculated as

F-score=$\frac{2*PPV*sensitivity}{PPV+Sensitivity}$

We also combined “Definite” and “Probable” as one single group to calculate and report these performance measurements as well for each feature.

**Reference**

1. Chapman WW, Bridewell W, Hanbury P, Cooper GF, Buchanan BG. A simple algorithm for identifying negated findings and diseases in discharge summaries. J Biomed Inform. 2001;34:301-310.
2. Chapman B, Chapman WW, Dayton G, Mowery D. Python Implementation of the ConText Algorithm. Available from: https://pypi.org/project/pyConTextNLP/. [Last accessed on 2019 Oct 02].
