# Supplementary material for: Characterization of patients with advanced chronic pancreatitis using natural language processing of radiology reports
Source: PLoS One. 2020 Aug 19;15(8):e0236817. doi: 10.1371/journal.pone.0236817 (PMC7437899; doi:10.1371/journal.pone.0236817)
Supplement: S1 Table — (DOCX) [file pone.0236817.s002.docx]

Table 1. Terms or phrases modifiers and exclusions used to identify the pancreatic features from radiology imaging reports.

| **Pancreatic features** | **Terms or phrases** | **Modifiers*** | **Exclusions*** |
| --- | --- | --- | --- |
| atrophy | atrophy, atrophic, atrophied |  |  |
| calcification | calcification, calcified, calcific |  |  |
| pseudocyst | pseudocyst^¶^, fluid collection |  |  |
| cyst | cyst |  | pseudo cyst, pseudo-cyst, cystic duct |
| ductal dilatation | dilated, dilation, dilatation, enlarge, enlarged, prominent, prominence | duct, ductal | bile duct, common duct, duct side branch, nondilated, nondilation |

* The plural of these terms is also included.

¶ Also includes pseudo cyst and pseudo-cyst.

Table 2. Distribution of the pancreas imaging features within the implementation dataset by the level of classification.

| Features | Classification level | |
| --- | --- | --- |
|  | Report (n,%) | Patient (n,%) |
| Atrophy  Definite  Probable  No | 2,802 (5.02%)  71 (0.13%)  52,959 (94.85%) | 1,222 (19.59%)  19 (0.30%)  5,057 (80.11%) |
| Calcification  Definite  Probable  No | 6,859 (12.29%)  189 (0.34%)  48,784 (87.38%) | 1,705 (27.33%)  40 (0.64%)  4,493 (72.03%) |
| Pseudocyst  Definite  Probable  No | 5,240 (9.39%)  1,380 (2.47%)  49,212 (88.14%) | 1,195 (19.16%)  301 (4.83%)  4,742 (76.02%) |
| Cyst  Definite  Probable  No | 3,677 (6.59%)  470 (0.84%)  51,685 (92.57%) | 1,254 (20.10%)  125 (2.00%)  4,859 (77.89%) |
| Ductal dilatation  Definite  Probable  No | 5,685 (10.18%)  138 (0.25%)  50,009 (89.57%) | 1,667 (26.72%)  37 (0.59%)  4,534 (72.68%) |

Table 3. The number of presented “Definite” or “Probable” pancreatic imaging features of atrophy, calcification, cyst, pseudocyst and ductal dilation in the implementation dataset by level of classification.

| Number of positive pancreatic imaging features | Classification level | |
| --- | --- | --- |
|  | Report (n,%) | Patient (n,%) |
| 0 | 37,618 (67.38%) | 2,741 (43.94%) |
| 1 | 12,103 (21.68%) | 1,321 (21.18%) |
| 2 | 4,307 (7.71%) | 956 (15.33%) |
| 3 | 1,464 (2.62%) | 692 (11.09%) |
| 4 | 298 (0.53%) | 384 (6.16%) |
| 5 | 42 (0.08%) | 144 (2.31%) |
