# Supplementary material for: Characterization of patients with advanced chronic pancreatitis using natural language processing of radiology reports
Source: PLoS One. 2020 Aug 19;15(8):e0236817. doi: 10.1371/journal.pone.0236817 (PMC7437899; doi:10.1371/journal.pone.0236817)
Supplement: S1 Error analysis — (DOCX) [file pone.0236817.s003.docx]

**Error analysis between the NLP algorithm and manual review against the validation dataset**

The discrepancies that we identified between the NLP algorithm and manual review were due to multiple factors. First, there were some cases classified by the NLP algorithm as “Definite”, but were assessed by manual review as “Probable” due to ambiguity in the dictated free text. For example, the status of cyst in the sentence “numerous additional calcifications in the pancreas with numerous cystic changes or dilated ducts in the pancreas” was classified as “Definite” by the computerized algorithm while it was classified as “Probable” by chart review. Second, there were several cases without the study designated feature terms/phrases, but through manual review the finding was able to be identified through the interpretation of the imaging report. For example, a “heterogenous collection containing both solid and liquid component” could be interpreted as a cyst although would not be recognized through any of the assigned keywords. Third, there were several false negative misclassifications due to the feature term associated with multiple organs. The current algorithm assumed the term only for the close proximity of organ. If the closer one was not pancreas, it was classified as “No”. For example, the sentence of “there is dilatation of the intrahepatic and of the pancreatic ducts” was misclassified as “No” for “ductal dilatation” status by the computerized algorithm because “intrahepatic” was closer to “dilatation”. Fourth, the current algorithm didn’t distinguish features as external versus internal to the pancreas, such as, “scattered internal calcifications” was misclassified as “Definite” for calcification by the NLP algorithm. Fifth, several cases of pseudocyst or cyst were related procedure notes rather than the imaging findings, such as “Imp: s/p pancreatic pseudocyst drain removal”. In addition, the computerized algorithm misclassified several cases because of the reference to historical events. For example, “the previously described cystic lesions adjacent to the pancreatic body and tail are again noted”. Finally, there are potentially clinically relevant explanations for lack of performance in certain characteristics. For example, the accuracy of the algorithm for detection of cyst features lagged compared with the other four features. This could be potentially related to ambiguity regarding the presence of a true pancreatic cyst versus an inflammatory pseudocyst when the images were being initially assessed for routine clinical purposes.
